# Assessing bacterial diversity and antibiotic resistance dynamics in wastewater effluent-irrigated soil and vegetables in a microcosm setting

**DOI:** 10.1016/j.heliyon.2022.e09089

**Published:** 2022-03-11

**Authors:** Onthatile Onalenna, Teddie O. Rahube

**Affiliations:** Department of Biological Sciences & Biotechnology, Faculty of Science, Botswana International University of Science & Technology, Palapye, Botswana

**Keywords:** Wastewater effluent, Antibiotic resistant genes, Agriculture, Metagenomics, Microcosm

## Abstract

Water scarcity is one of the main challenges in sustainable agricultural development particularly in developing countries therefore, irrigation of food crops with wastewater effluent has become a common practice in order to meet the growing food demand. The aim of this study was to determine the impact of wastewater irrigation on bacterial community and antibiotic resistance dynamics in soil and vegetables in an agricultural setting. To determine bacterial diversity, occurrence and overall dynamics of antibiotic resistant genes (ARGs) in effluent irrigated soil and vegetables, 16S rRNA gene metagenomics, shotgun metagenomics and molecular PCR technique were utilized. A shift in bacterial community profile was observed as notable reduction in proteobacteria and increase in firmicutes phyla from the microcosm soil following wastewater effluent irrigation. Shotgun metagenomics revealed diverse ARGs belonging to at least nine different classes of antibiotics in the effluent wastewater. However, only *bla*_TEM_ (beta-lactamase) and *aad*A (aminoglycoside) resistance gene sequences were identified in microcosm soil following irrigation and only *bla*_TEM_ was detected on effluent irrigated vegetable surfaces (spinach and beetroots). From the study, only *bla*_TEM_ gene was identified across all samples; effluent wastewater, effluent-treated soil, and vegetables. The data suggests a possible dissemination and persistence of the beta-lactamase *bla*_TEM_ gene from effluent wastewater into agricultural soil and vegetables. This study enhances our understanding of antibiotic resistance spread and highlights the importance of monitoring antibiotic resistance in agro-systems, which is critical for informing policies aimed at sustainable use of wastewater effluent in water-stressed countries.

## Highlights

• Wastewater effluent potentially introduces antibiotic resistance genes into the soil and vegetables.
• The beta-lactamase (*bla*_TEM_) gene from effluent wastewater is potentially introduced into agricultural soil and vegetables.
• Monitoring antibiotic resistance in agro-systems is critical for informing policies aimed at sustainable use of wastewater.
• Irrigation with wastewater effluent remains unregulated in most developing countries.

## 1 Introduction

The global use and misuse of antibiotics have resulted in the accumulation of antibiotic resistant bacteria (ARB) and antibiotic resistance genes (ARGs) in the environment which has become an inevitable global public health threat. WWTPs are considered propagation routes for antibiotic resistance determinants due to antibiotic residues, high bacteria density and nutrient content in sewage. WWTPs collects waste from different environments such as households, industries and health care services, this has therefore resulted in them being involuntary accumulation points for antibiotics, ARB and ARGs. Though WWTPs differ in design and processes they assemble three successive steps; pre-treatment, primary treatment and secondary treatment (Grady et al., 2011). Wastewater treatment processes are not aimed at removing ARB and ARGs, so the effluent released may consist of antibiotic resistant determinants that are therefore released into the environment (Berendonk et al., 2015). The composition of sewage microbiota is primarily environmental bacteria and human commensal bacteria (Shchegolkova et al., 2016). It is therefore expected that wastewater reflects characteristics of the human microbiome.

Irrigation of crops using wastewater effluent has been adapted by many African countries such as South Africa, Tunisia, Zimbabwe and Botswana, this reduces the need for fresh water while improving food security (Khalid et al., 2018; Onalenna and Rahube 2019). Increasing reports have shown that plants have the ability to passively uptake water soluble contaminants through the roots which can be translocated and concentrated into other parts of the plant such as leaves, although this more so in hydroponic cultures compared to conventional crops in soil (Pullagurala et al., 2018; Madikizela et al., 2018). Uptake of ARB and ARGs by plants is determined by several factors including, the physiochemical properties of the contaminant, the plant genotype, physiological state of the plant and stress effects on the plant such as weather conditions (Madikizela et al., 2018).

Food safety is increasingly becoming a public health concern, fresh produce is an essential part of a healthy diet as they provide nutrients such as vitamins, minerals, antioxidants and fiber hence it has become a preferred option as more people are becoming health conscious (Iwu and Okoh 2019). The global consumption of fresh produce has significantly increased, from ∼10g to ∼110g in individuals per day in sub-Saharan Africa (Mensah et al., 2020). Fruits and vegetables irrigated with effluent are highly exposed to microbial contamination through contact with effluent irrigated soil and wastewater effluent. Some leafy green vegetables require no heat treatment before consumption therefore there is an increased risk of ARB and ARGs exposure to humans through consumption of fresh produce (Holvoet et al., 2013). Antibiotic resistance pool in the human gut can be increased by multi-drug resistant (MDR) bacteria that are carried by raw vegetables (Walia et al., 2013). This therefore elevates the likelihood of plasmid conjugal transfer between bacteria on vegetables and human gut flora (Schjørring and Krogfelt 2011). The emergence of antibiotic resistance in vegetables is proving to be a serious concern affecting human and environmental health. Previous studies have revealed the abundance of antibiotic resistant coliforms and pathogenic bacteria as well as persistence of ARGs linked to wastewater/sewage isolated from soil and vegetables at harvest as well as in retail (Kilonzo-Nthenge and Mukuna 2018, Rahube et al., 2014, 2016).

Human health implications of consuming produce with ARB and ARGs is mostly unknown. The question then becomes whether ARB from the environment and that in the human gut consists of a common pool. It has been speculated that ARGs cause potential health impacts such as disrupted digestive system functions, allergic reactions and chronic toxic effects (Berglund et al., 2014). Even at low abundance these bacteria may be transmitted to humans in an asymptomatic long-term colonization which may only surface when the immunity is compromised (Christou et al., 2017).

The Government of Botswana has implemented interventions as part of the vision 2036 pillar for sustainable economic development towards food security (Mogomotsi et al., 2018). Horticultural farmers are allocated land near WWTPs to use wastewater effluent to cultivate fresh produce that can be supplied to government schools in an effort to combat under and malnutrition. Produce from these farms are also supplied to local supermarkets which empowers the farmers and contribute to the country's food security. The Government of Botswana also encourages its citizens to practice backyard gardening through one of its poverty eradication programs (Marumo et al., 2017). There is little research conducted on the environmental dimension of antibiotic resistance in Botswana, and these government initiatives present a potential risk for antibiotic resistance dissemination in agricultural environments and potentially to humans through consumption of contaminated vegetables.

## 2 Materials and methods

### 2.1 Description of study area

#### 2.1.1 Microcosm experiment

The government of Botswana has introduced an irrigation scheme where land is allocated to horticulture farmers around wastewater treatment plants and wastewater effluent is used as a source of irrigation water. This scheme has been introduced to Palapye, a peri-urban rapidly growing town with prominent rural lifestyle and population around 36,000 people. Palapye wastewater treatment plant (PWWTP) has a receiving influent daily capacity of 14000m^3^ and uses pond enhancement treatment for anaerobic digestion. The final effluent from PWWTP is chlorinated then discharged into a manmade pond located downstream of the treatment facility. PWWTP effluent is the main source of water used for various purposes such drinking by livestock, construction and irrigation of fresh produce in backyard gardens by the local community. Mahibitswana, located approximately 1km from PWWTP is a proposed agricultural field used for the Government irrigation scheme that uses PWWTP effluent as a source of irrigation water. This field was used as source of soil samples that were collected for the microcosm experiment.

#### 2.1.2 Microcosm experimental design

**Figure 1:**
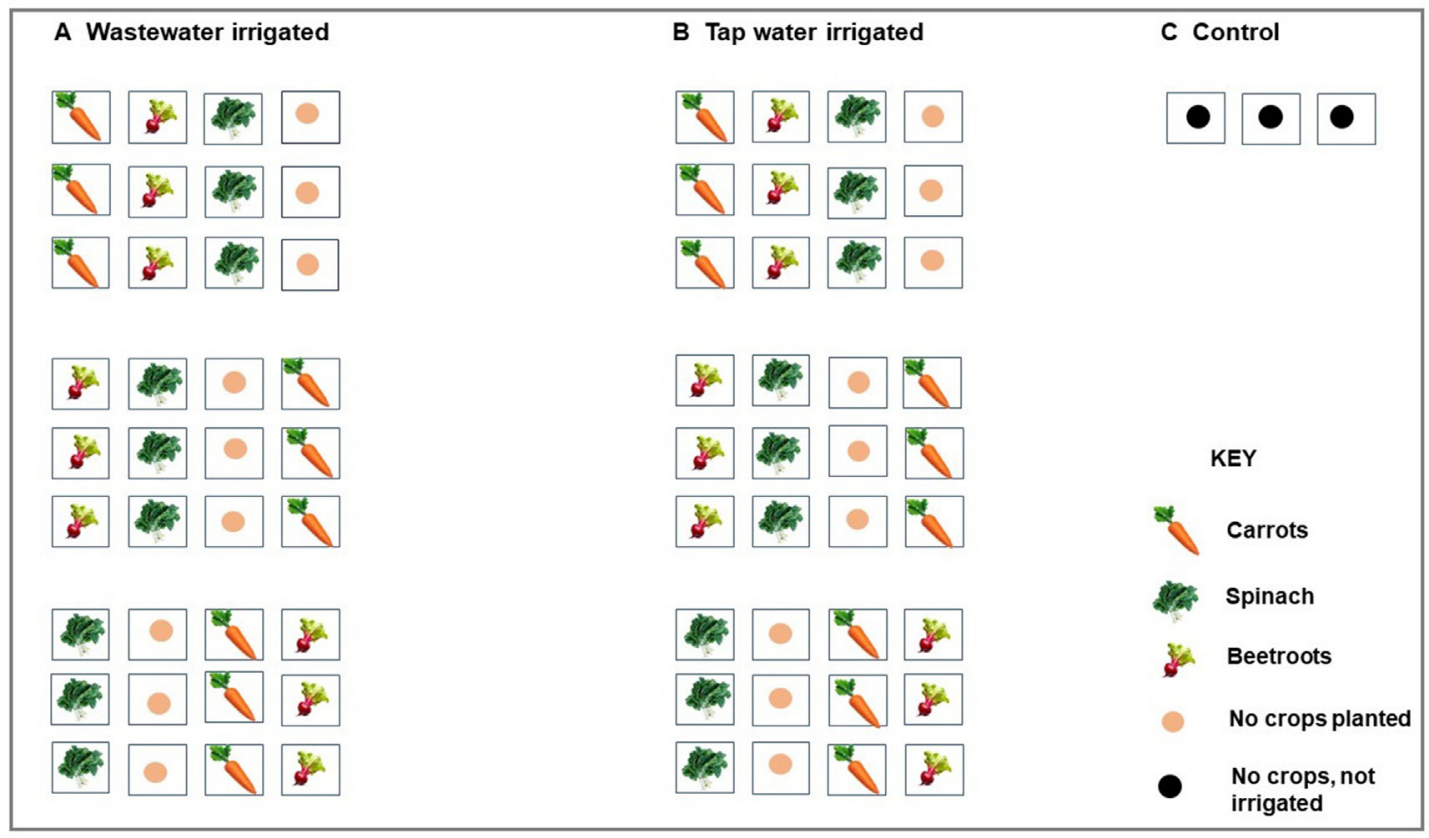
Microcosm experimental design. A; Experimental microplots showing vegetables that were sown and irrigated using PWWTP effluent. B; Control microplots showing vegetables that were sown and irrigated using tap water. C: Untreated microplots.

Soil samples were collected into sterile 5L black planting bags filling up to 70% of the bag (∼5kg). Wastewater effluent from PWWTP was filled into a 2500L plastic water storage tank and used as a source of water for irrigation of the microcosm garden. Two microcosm experiments were set comprising spinach (*Spinacia oleracea*), beetroots (*Beta vulgaris*) and carrots (*Daucus carota subsp. Sativus*) that were sown directly in the soil, one set of the microcosm (A) was irrigated with wastewater effluent (experimental), another set (B) was irrigated with tap water (control). An additional set of three planting bags (C) were not sown and not irrigated (untreated) throughout the course of 90 days experiment (Figure 1). In order to minimize cross contamination during the course of the experiment, set A was 2 m apart from set B, which was 1.25m apart from set C. To mimic the local backyard gardening commonly practiced in local communities, the soil was kept moist with routine irrigation every 2 days (with approximately 1.5 L of water going into each planting bag), weeds were removed aseptically by hand, other external environmental factors such as wind, temperature and humidity were not controlled. The experiment was conducted between June and October, which was mostly sunny with temperatures ranging from 17 °C to 39 °C.

### 2.2 Sample collection

Sampling was conducted starting July 2018 (for 3 months). From the effluent wastewater in the storage tank, 1L was collected into a sterile polystyrene bottle at day 1 (immediately after filling the tank) and every month (30 days) after for 3 months. Soil samples (5g) were collected at a depth of 15cm in triplicates into sterile zip lock bags from each plating bag at the beginning of the experiment before irrigation and every 30 days post irrigation for 3 months. The samples were immediately analyzed in the laboratory. All spinach leaves and beetroots were aseptically harvested (40 days post sowing for spinach and 60 days for beetroots) into sterile zip lock bags and immediately taken to the lab for analysis. Due to the extreme temperature carrots did not grow therefore only spinach and beetroots were harvested for analysis.

### 2.3 DNA extraction

DNA was extracted from the samples within 4 h of collection. Briefly 500ml of wastewater effluent from PWWTP and vegetable wash water were filtered through a 0.45μm filter paper and DNA was extracted using the ZR microbe DNA extraction kit (Zymo Research USA) following manufacturer's instructions. DNA from effluent irrigated soil samples, tap water irrigated soil samples and untreated soil samples was extracted in triplicates following DNA extraction protocol using the ZR microbe DNA extraction kit (Zymo Research USA) following manufacturer's instructions. The yield of the extracted DNA was quantified and checked for purity using a nano drop spectrophotometer (Lasec, Jenway Genova nano) at an absorbance of 260nm. All the DNA samples obtained were stored at -20 °C for further analysis.

### 2.4 16S rRNA gene sequencing

Uncultured and community DNA from wastewater effluent and wastewater effluent irrigated soil were analyzed by 16S rRNA gene sequencing on the Illumina MiSeq system following a workflow by Klindworth et al. (2013). The protocol included genomic DNA being PCR amplified using a universal primer pair 341F and 785R - targeting the V3 and V4 region of the 16S rRNA gene. Amplicons were then purified by gel, end repaired and illumina specific adapter sequence were ligated to each amplicon. The samples were quantified and individually indexed followed by another purification step. Amplicons were then sequenced on Illumina's MiSeq platform, using a MiSeq v3 (600 cycle) kit. Sequence reads were processed through research (https://drive5.com/usearch) and taxonomic information was determined based on the Ribosomal Database Project's (http://rdp.cme.msu.edu/index.jsp) 16S database v16 or in the case of ITS1F, the RDP ITS V2 database.

### 2.5 Shotgun metagenomics

Uncultured and community DNA from wastewater effluent and wastewater effluent irrigated soil samples were fragmented using an enzyme-based approach following part of the protocol from New England BioLab's Next Ultra II kit™. Resulting fragments were purified (size selected), end-repaired and an Illumina specific adapter sequence was ligated to all fragments.The samples were quantified, individually indexed followed by a second size selection step using AMPure XP Beads. The libraries were quality controlled on a DNA chip (Agilent 2100 Bioanalyzer) and then sequenced on Illumina's MiSeq platform, using a MiSeq v3 (600 cycle) kit according to the manufacturer's protocol.

### 2.6 Data processing and statistical analysis

SPAdes was used through PATRIC (https://www.patricbrc.org/) to assemble reads obtained from illumina shotgun metagenomics sequencing. Annotation of contigs was carried out with Rapid Annotation using Subsystem Technology (RAST) tool kit (http://rast.theseed.org/FIG/rast.cgi), PATRIC k-mer based tool (https://www.patricbrc.org/) was used to assign ARG functional annotation and broad antibiotic resistance mechanisms. ResFinder (https://cge.cbs.dtu.dk/services/ResFinder/) and CARD (https://card.mcmaster.ca/) were used to identify acquired ARGs. Adonis test was performed in QIIME 2 to identify differences in community composition between wastewater-treated and untreated soil samples. The statistical test was considered significant at P-value < 0.05.

### 2.7 Data availability

The sequence data is available at the NCBI SRA under the Bioproject Accession: PRJNA797192: https://www.ncbi.nlm.nih.gov/sra/?term=PRJNA797192.

### 2.8 Polymerase chain reaction (PCR) assay

Conventional PCR was used for the detection three ARGs; *bla*_TEM_ (**F**- TCCGCTCATGAGACAATAACC, **R**- TTGGTCTGACAGTTACCAATGC), *dfr*A (**F**-CCCAACCGAAAGTATGCGGTCG, **R**-GTATCTACTTGATCGATCAGG), and *aad*A (**F-** GTGGATGGCGGCCTGAAGCC, **R**- AATGCCCAGTCGGCAGCG) conferring resistance to beta-lactam, trimethoprim and aminoglycosides respectively in the effluent wastewater, soil and vegetable DNA samples (Asir et al., 2015; Tapela and Rahube 2019; Titilawo et al., 2015). All three target genes were previously detected in effluent wastewater, the DNA from effluent wastewater was used as positive control for PCR assay. PCR assays with positive control and negative control (nuclease free water) consisted of a total reaction volume of 25μl which constituted of 12.5μl Emerald Amp® GT PCR Master Mix, 1.5 μl each primer (10μM), 7.5μl nuclease free water and 2μl DNA template (ranging from 0.1ng to 10ng). Target genes were amplified in a conventional PCR machine (ProFlex PCR system), the temperature profile entailed initial denaturation of 95 °C for 5 min, followed by 35 cycles of 98 °C for 10 s, 1-min annealing at specific primer temperatures, 72 °C for 1 min with a final extension at 72 °C for 1 min. The annealing temperatures are specified in Table 2.4.1. PCR products were analyzed by 1% (w/v) agarose gel electrophoresis stained in 4 μl/g ethidium bromide for 90 min in 1× TAE buffer and viewed using UV light (Gel doc-IT® imager UVP, Cambridge, UK). The sizes of the PCR products were confirmed against Quick-Load 1 kb DNA ladder (BiLabs inc, England and confirmed by DNA sequencing.

## 3 Results

### 3.1 Bacterial communities in effluent, effluent-treated and untreated soil

Metagenomics sequencing of the16S rRNA gene was carried out to determine the diversity of bacterial phylogenetic groups in PWWTP effluent, effluent-treated and untreated soil. From the PWWTP effluent, Cyanobacteria phylum was over-represented with 48% followed by Firmicutes (21%), Proteobacteria (17%), Actinobacteria (13%) and Bacteroidetes (1%). At class level Oscillatoriophycideae showed a high percentage of 43%, Bacilli (20%), Gammaproteobacteria (14%), Actinobacteria (13%), Betaproteobacteria (2%), Alphaproteobacteria (1%), and Bacteroidia was least represented with 1% in the total bacterial population of PWWTP effluent sample. Bacteria identified were classified into different genus; *Streptococcus* (18%), *Pasteurella* (8%), *Rothia* (8%), *Enterobacter* (5%), *Pseudomonas* (3%), *Escherichia* (2%), *Actinomyces* (2%), *Neisseria* (2%), *Stella* (1%) and *Salmonella* (1%).

From the PWWTP effluent-treated soil sample, Actinobacteria was the most abundant phylum with 42% representation, followed by Proteobacteria with 22%. Firmicutes (14%), Plantomycetes (10%), Acidobacteria (2%), Chloroflexi (3%), Bacteroidetes (2%) and Gemmatimonadetes (2%) were also identified. At class level, Actinobacteria was over-represented with 34% followed by Alphaproteobacteria (12%), Bacilli (11%), Planctomycetia (10%), Gammaproteobacteria (5%), Betaproteobacteria (4%). Ktedonobacteria accounted for 3%, Clostridia 2% and Deltaproteobacteria 1%. Out of all the identified bacterial species, 15% were classified as *Streptococcus*, 11% as *Bacillus*, *Conexibacter* 7% and *Solurobacter* 6%. *Streptomyces* and *Methylobacterium* each accounted for 5% of the species identified, *Escherichia* had 3% representation. *Salmonella* and *Neisseria* were the least represented genus with 1% each genus (Table 1).

**Table 1:** Summary of bacterial diversity and abundance in PWWTP effluent, effluent-treated soil and untreated soil.

	PWWTP effluent	Cyanobacteria (48%), Firmicutes (21%), Proteobacteria (17%), Actinobacteria (13%), Bacteroidetes (1%)
Phylum	Effluent-treated soil	Actinobacteria (42%), Proteobacteria (22%), Firmicutes (14%), Plantomycetes (10%), Chloroflexi (3%), Acidobacteria (2%), Bacteroidetes (2%), Gemmatimonadetes (2%)
	Untreated soil	Proteobacteria (88%), Firmicutes (5%), Actinobacteria (4%), Plantomycetes (2%), Bacteroidetes (1%)
	PWWTP effluent	Oscillatoriophycideae (43%), Bacilli (20%), Gammaproteobacteria (14%), Actinobacteria (13%), Betaproteobacteria (2%), Alphaproteobacteria (1%), Bacteroida (1%)
Class	Effluent-treated soil	Actinobacteria (34%), Alphaproteobacteri (12%), Bacilli (11%), Gammaproteobacteria (5%), Plancomyceti (10%), Betaproteobacteria (4%), Ktedobacteria (3%), Clostridia (2%), Deltaproteobacteria (1%)
	Untreated soil	Gammaproteobacteria (79%), Comamonas aquatica (9%)
	PWWTP effluent	Lactobacillales (19%), Micrococcales (9%), Pasteurellaceae (8%), Enterobacterales (4%), Pseudomonadales (2%), Actinomycetaceae (2%)
Order	Effluent-treated soil	Bacillales (11%), Micrococcales (6%), Rhizobiales (5%), Streptosporangiales (4%), Crynebacterales(4%), Lactobacillales (4%), Rhodospirillales (4%), Burkholderales (4%), Streptomycetaceae (3%), Enterobacterales (1%)
	Untreated soil	Enterobacterales (43%), Pseudomonadales (27%)
	PWWTP effluent	Streptococcaceae (18%), Micrococcaceae (8%), Enterobacteriacea (3%), Pseudomanadaceae (2%)
Family	Effluent-treated soil	Bacillacea (4%), Streptococcacea (4%), Methylobacteriacea (2%), Enterobacteriacea (1%), Micrococcaceae (1%)
	Untreated soil	Enterobacteriacea (34%), Pseudomonadaceae (25%)
	PWWTP effluent	Streptococcus (18%), Pasteurella (8%), Rothia (8%), Enterobacter (5%), Pseudomonas (3%), Escherichia (2%), Actinomycetes (2%), Neisseria (2%), Stella (1%), Salmonella (1%)
Genus	Effluent-treated soil	*Streptococcus* (15%), *Bacillus* (11%), *Conexibacter* (7%), *Solurobacter* (6%) < *Streptomyces* (5%), *Methylobacterium* (5%), *Escherichia* (3%), *Salmonella* (1%), *Neisseria* (1%)
	Untreated soil	*Pseudomonas* (25%), *Comamonas* (9%), *Proventia* (9%), *Escherichia* (7%), *Klebsiella* (7%), *Citrobacter* (7%), *Streptococcus* (5%), *Enterobacter* (2%), *Acinetobacter* (2%)

Five phyla were identified in untreated soil sample; Proteobacteria (88%), Firmicutes (5%), Actinobacteria (4%), Planctomycetes (2%) and Bacteroidetes (1%). From these phylum Gammaproteobacteria was shown to be the highest class with 79% representation, with Comamonas aquatica representing 9% of the total bacteria. At genus level, 25% of the bacteria classified were *Pseudomonas*, *Comamonas* and *Provencia* had 9% representation each genus, *Escherichia*, *Klebsiella* and *Citrobacter* each had 7% representation. *Streptococcus* was represented with 5% and *Enterobacter* and *Acinetobacter* each 2% of the total bacteria from untreated soil sample (Table 1).

Obvious difference in community composition of PWWTP effluent-treated soil and untreated soil was observed (Adonis test p < 0.05) likely indicating the impact of effluent irrigation on soil community structure.

Proteobacteria and Firmicutes phyla were compared between PWWTP effluent, effluent-treated and untreated soil samples to determine the effects of effluent wastewater on the bacterial communities in the soil before and after irrigation.

**Figure 2:**
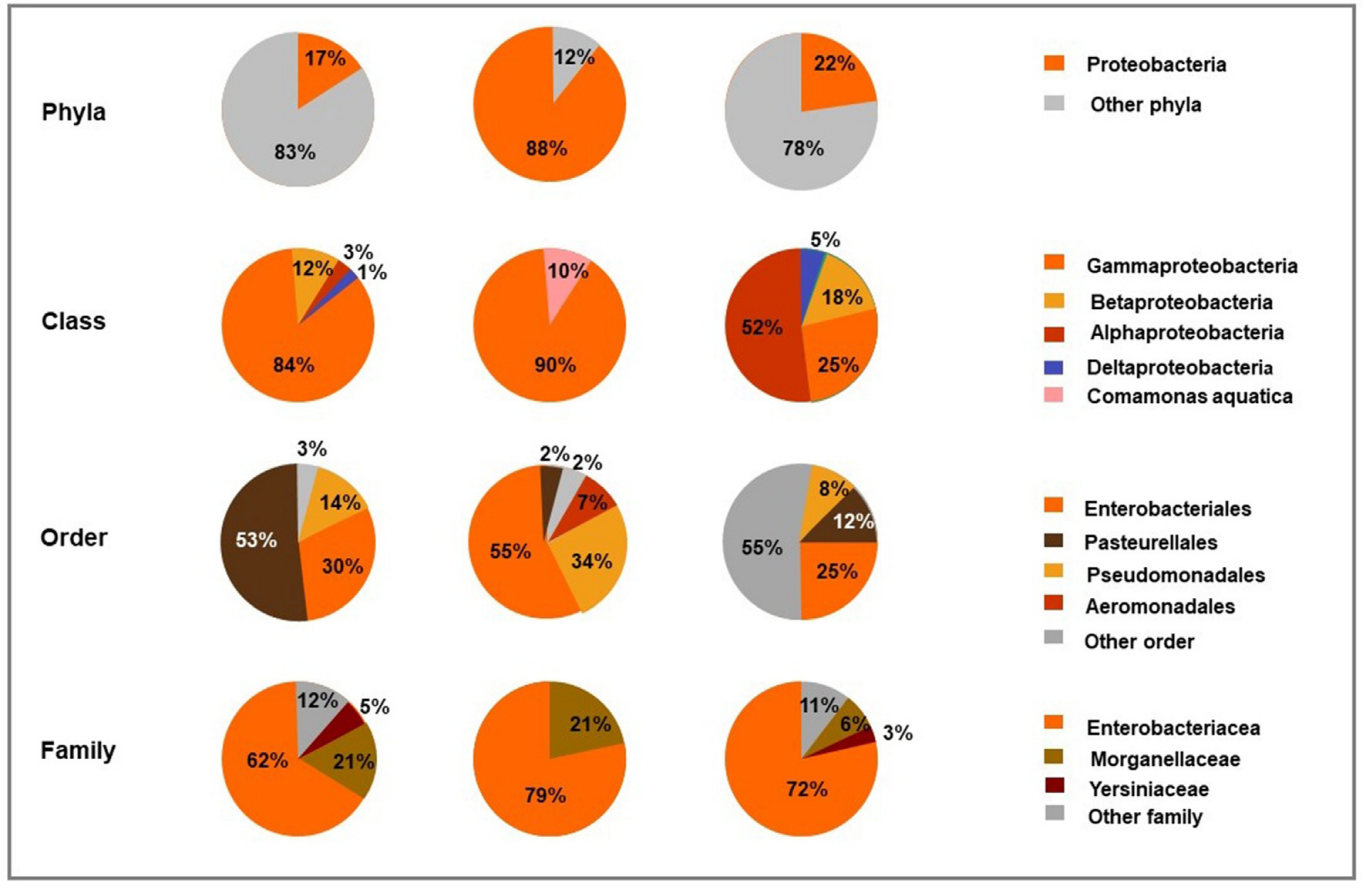
Taxonomic classification comparison based on Proteobacteria found in PWWTP effluent, untreated soil and effluent irrigated soil.

Proteobacteria was identified in PWWTP effluent, untreated soil and 90 days effluent-treated soil (Figure 2). At phyla level a reduction in Proteobacteria was observed from 88% (in untreated soil) to 22% following irrigation with wastewater effluent. A notable reduction in Proteobacteria is further observed at class level with 90% Gammaproteobacteria in untreated soil and 25% in effluent-treated soil where 84% Gammaproteobacteria was observed in PWWTP effluent. Other bacteria classes such as Betaproteobacteria, Alphaproteobacteria and Deltaproteobacteria are seen to be introduced into the soil since they were not identified in untreated soil. At order level Enterobacteriales and Pseudomonadales also reduced from 55% and 34% in untreated soil to 25% and 8% respectively in effluent-treated soil. At family level Enterobacteriacea slightly reduced from 79% in untreated soil to 72% in effluent-treated soil where 62% Enterobacteriacea were observed in PWWTP effluent. Yersiniaceae and Erwiniaceae also appear to be introduced from effluent into soil after irrigation, since these bacterial families were not observed in untreated soil.

**Figure 3:**
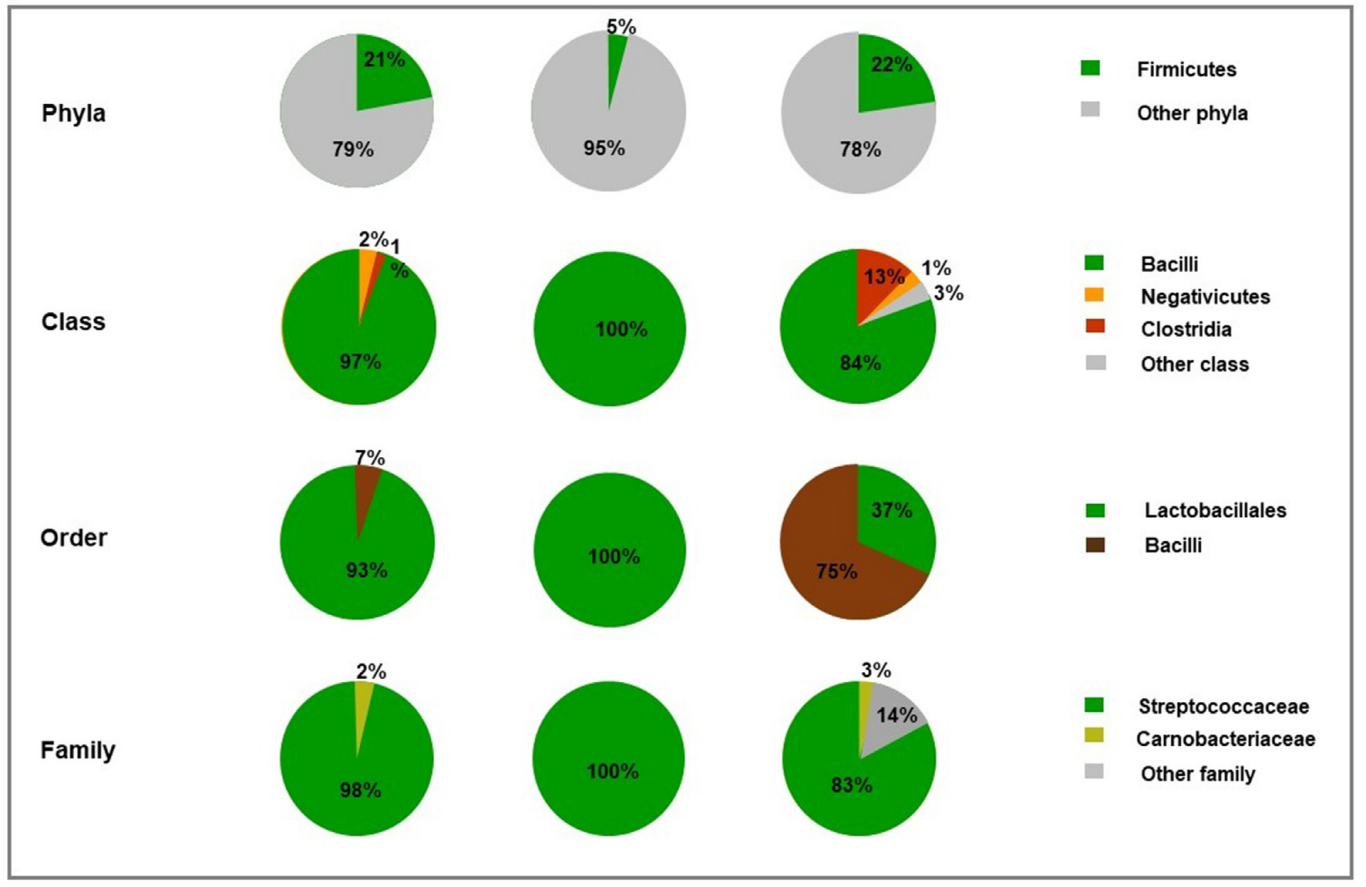
Taxonomic classification comparison based on Firmicutes found in PWWTP effluent, untreated soil and effluent irrigated soil.

Firmicutes were identified in PWWTP effluent, untreated soil and 90 days effluent-treated soil. An increase in firmicutes phyla was observed in soil as 5% was identified in untreated soil and 14% in PWWTP effluent-treated soil with 21% firmicutes observed in PWWTP effluent wastewater. At class level, only bacilli (100%) were observed in untreated soil whereas 84% was observed in effluent-treated soil and 97% in PWWTP effluent wastewater. Negativicutes and Clostridia were identified in both PWWTP effluent and effluent-treated soil. At order level only Lactobacillales were identified in untreated soil, a notable reduction was observed in effluent-treated soil with 37% Lactobacillales and 75% Bacilli of which PWWTP effluent had 7% Bacilli. Lactobacillaceae (8%), Leuconostacaceae (3%) and Enterococcaceae (3%) were observed in effluent-treated soil but not in PWWTP effluent and untreated soil (Figure 3).

### 3.2 Shotgun metagenomic analysis

Using ResFinder and CARD, diverse acquired ARGs were identified in PWWTP effluent and classified under several clinically important classes of antibiotics; aminoglycosides, beta-lactamase, trimethoprim, macrolide, glycopeptide, tetracycline, sulfonamides, quinolones and oxazolidinone. Specific antibiotic inactivation mechanisms were identified associated with aminoglycosides (*aad*A5, *aac(2)-*la*, aph(6)-*Id, *aph (3)-*Ib), beta-lactamase (*bla*_TEM_, *bla*_CTX-M,_ *bla*_TEM -122,_ *bla*_SHV-163,_ *bla*_OXA__*-663,*_ *ampC*) and macrolide (*mph*A) genes. *Beta*-lactamase *ompk35* gene was associated with conferring resistance through a different mechanism that reduces permeability to antibiotics. Trimethoprim (*dfr*A1*, dfr*A14, *dfr*A17), sulfonamides (*sul*1, *sul*2*, sul*3) and some quinolones (*qnr*B5*, qnr*B10*, qnr*S1*, qnr*D1*, qnr*D2) ARGs were identified and these triggers resistance through modification of antibiotic targets. The glycopeptides (*tol*C*, acr*A, *acr*B, *acr*D, *acr*F, *cpx*A), tetracycline (*gad*W, *gad*X*, tet*(A), *tet*(B), *tet*(C), *tet*(D)*, tet*(R)*, tet*(39)*, evg*S), some quinolones (*emr*A, *emr*B, *emr*K, *emr*Y) and oxazolidinome (*mdt*B, *mdt*F*, mdt*K, *mdt*H, *mdt*O, *mdt*P) genes were identified and confer resistance through the antibiotic efflux mechanism (Table 2).

**Table 2:** Acquired ARGs detected in PWWTP effluent and PWWTP effluent-treated soil.

Antibiotic class	ARGs	% identity	% Length of Reference sequence	Resistance Mechanism
Aminoglycosides	*aad*A5	100	19.85	Antibiotic inactivation
	*aac(2)-*la	99.08	61.24	Antibiotic inactivation
	*aph(6)-*Id	99.64	100	Antibiotic inactivation
	*aph(3)-*Ib	100	13.45	Antibiotic inactivation
*Beta*-lactamases	*bla*_TEM_	100	94.06	Antibiotic inactivation
	*bla*_CTX-M_	100	64.21	Antibiotic inactivation
	*bla*_TEM -122_	100	94.06	Antibiotic inactivation
	*bla*_SHV-163_	97.18	36.36	Antibiotic inactivation
	*bla*_OXA__*-663*_	100	84.21	Antibiotic inactivation
	*ampC*	100	8.29	Antibiotic inactivation
	*omp*k35	100	21.93	Reduced permeability to antibiotic
Macrolides	*mph*A	100	100	Antibiotic inactivation
Trimethoprim	*dfr*A1	99.36	100	Antibiotic target replacement
	*dfr*A14	100	100	Antibiotic target replacement
	*dfr*A17	99.07	58.15	Antibiotic target replacement
Glycopeptides	*tol*C	99.51	4.41	Antibiotic efflux
	*acr*A	98.64	17.21	Antibiotic efflux
	*acr*B	96.18	12.58	Antibiotic efflux
	*acr*D	95.52	5.45	Antibiotic efflux
	*acr*F	100	4.45	Antibiotic efflux
	*cpx*A	100	24.29	Antibiotic efflux
Tetracycline	*gad*W	100	10.27	Antibiotic efflux
	*gad*X	94.21	17.19	Antibiotic efflux
	*tet*(A)	100	97.88	Antibiotic efflux
	*tet*(B)	100	5.99	Antibiotic efflux
	*tet*(C)	99.51	22.47	Antibiotic efflux
	*tet*(D)	100	40.86	Antibiotic efflux
	*tet*(R)	100	28.85	Antibiotic efflux
	*tet*(39)	100	22.28	Antibiotic efflux
	*evg*S	99.68	56.22	Antibiotic efflux
Sulfonamides	*sul*1	100	100	Antibiotic target replacement
	*sul*2	100	100	Antibiotic target replacement
	*sul*3	100	100	Antibiotic target replacement
Quinolones	*qnr*B5	100	100	Antibiotic target protection
	*qnr*B10	99.12	100	Antibiotic target protection
	*qnr*S1	100	100	Antibiotic target protection
	*qnr*D1	100	100	Antibiotic target protection
	*qnr*D2	100	13.55	Antibiotic target protection
	*emr*A	100	7.42	Antibiotic efflux
	*emr*B	100	4.3	Antibiotic efflux
	*emr*K	97.67	12.82	Antibiotic efflux
	*emr*Y	100	18.34	Antibiotic efflux
Oxazolidinome	*mdt*B	100	2.50	Antibiotic efflux
	*mdt*F	96.77	4.34	Antibiotic efflux
	*mdt*K	96.3	5.70	Antibiotic efflux
	*mdt*H	99.21	31.59	Antibiotic efflux
	*mdt*O	98.55	20.20	Antibiotic efflux
	*mdt*P	100	2.90	Antibiotic efflux

Aminoglycoside (*aad*A6) gene associated with the antibiotic inactivation resistance mechanism was identified in effluent-treated soil. Only *bla*_TEM_ (beta-lactam) acquired resistant gene was observed in both effluent and effluent-treated soil in the microcosm experiment (Table 2).

### 3.3 Detection of antibiotic resistance genes in effluent-treated soil and vegetables using conventional PCR

**Figure 4:**
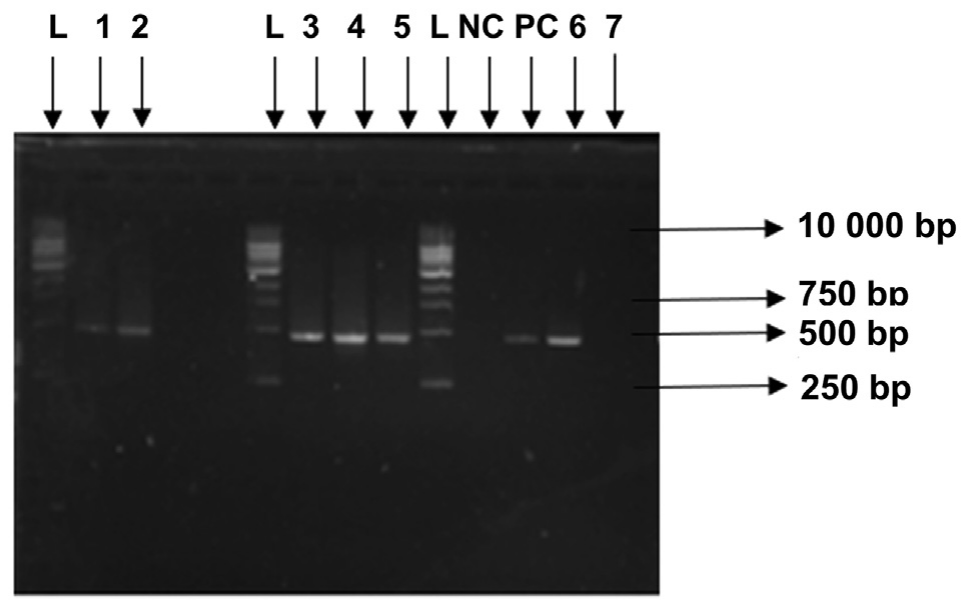
PCR amplification of targeted *bla*_TEM_ gene. Key – L; 1KB DNA Ladder, NC; Negative control, PC; Positive control, 1; Wastewater effluent irrigated soil after 30 days, 2; Wastewater effluent irrigated soil after 60 days, 3; Wastewater effluent irrigated soil after 90 days, 4; Spinach at harvest, 5: Beetroot at harvest, 6; Wastewater effluent 7; Untreated soil.

The occurrence of ARGs from the microcosm experiment; untreated soil, PWWTP effluent-treated soil and vegetables, was determined using conventional PCR. Gene target, *bla*_TEM_ was not identified in untreated soil, however was detected in wastewater effluent, 30, 60 and 90 days effluent irrigated soil and vegetable surfaces (Figure 4). Genes *dfr*A and *aad*A were not detected in untreated soil as well as wastewater treated soil and vegetables (Table 3).

**Table 3:** Antibiotic resistance genes detected in microcosm experiments using PCR.

Gene Target	Wastewater effluent	Untreated soil	Microcosm experiment			
			Soil 30 days post WW irrigation	Soil 60 days post WW irrigation	Wastewater irrigated spinach	Wastewater irrigated beetroot
*bla*_TEM_	+	-	+	+	+	+
*dfr*A	+	-	-	-	-	-
*aadA*	+	-	-	-	-	-

+ Presence, - Absence.

## 4 Discussion

Wastewater effluent remains an important source of irrigation water in many developing countries. However due to the poor infrastructure of wastewater treatment plants and lack of regulations on safe use of effluent, this water source potentially spread antibiotic resistance determinants in agricultural soil and vegetables posing a serious public health concern. This study was carried out to determine the impact of wastewater irrigation on the diversity and overall dynamics of bacterial communities and antibiotic resistance genes (ARGs) in agricultural settings.

### 4.1 Impact of wastewater irrigation on bacterial diversity in soil

Next generation sequencing of the 16s rRNA gene was carried out to determine bacterial diversity in PWWTP effluent, PWWTP effluent-treated soil and untreated soil. Proteobacteria was found to be the predominant phyla in untreated soil, with Gammaproteobacteria being the most abundant class of Proteobacteria. Gammaproteobacteria comprises of foodborne pathogens such as *Escherichia*, *Salmonella* and *Enterobacteriaceae*, Gammaproteobacteria is important in the global cycling of carbon, nitrogen and sulfur hence expected to be found in soil (Mhete et al., 2020). A reduction in gammaproteobacteria was observed in the soil following wastewater effluent irrigation, from 90% Gammaproteobacteria in untreated soil to 52% in effluent-treated soil. Previous studied carried out by Broszat et al. (2014) indicate that soil irrigated with wastewater for a period of 100 years in Mexico showed 26.7% increase in the relative abundance of Proteobacteria. It has also been reported that the relative abundance of Proteobacteria increases with high carbon availability in soil (Mhete et al., 2020). This however contradicts the results of this study since a reduction in Gammaproteobacteria was observed following wastewater irrigation. The microcosm study was carried out for 3 months and the overall trend of Proteobacteria may have not been completely captured during the short-term microcosm experiment.

Secondary wastewater treatment provides a conducive environment for growth of Cyanobacteria, proliferation of Cyanobacteria is then enhanced by increased light and high summer temperatures (Martins et al., 2011). This justifies the high abundance of Cyanobacteria in PWWTP effluent. The growth of Cyanobacteria in wastewater effluent drastically changes the ecology of the microbial communities. Moreover, the presence of Cyanobacteria in wastewater effluent may result in toxin production which presents a serious public health issue when disseminated to downstream environments (Martins et al., 2011). In PWWTP effluent-treated soil, Actinobacteria was found to be the most dominant phyla. Actinobacteria consists of many Gram-negative bacteria that play an important role in carbon cycling and degrading environmental chemicals. The results of this study are supported by Ouyang et al. (2017) who previously identified the phyla as the most prevalent in activated sludge and wastewater treated soils.

Following Cyanobacteria, Firmicutes were most abundant in PWWTP effluent. Firmicutes were identified in PWWTP effluent (21%), untreated soil (5%) and 90 days effluent-treated soil (14%). An increase in Firmicutes abundance is observed in wastewater-treated soil. This is expected because an increase in soil carbon content increases the nutritive value of soil hence increase in bacterial proliferation. An increase in firmicutes abundance in soil presents a public health issue as Firmicutes comprise of notable Gram-positive bacteria such *Clostridium, Streptococcus* and *Staphylococcus* species associated with causing diseases in humans (Mhete et al., 2020).

### 4.2 Dynamics of ARGs in soil and vegetables following wastewater irrigation

The pathway for transmission of ARGs from effluent and soil to vegetables is still not understood. From the shotgun metagenomic sequencing results of this study, antibiotic resistance genes for at least nine antibiotic classes were identified in PWWTP effluent. However only beta-lactamase and aminoglycoside genes were identified in both PWWTP effluent and effluent-treated soil, and only beta-lactamase gene (*bla*_*TEM*_) in vegetable surfaces. This may be attributed to the short-term irrigation of the microcosm experiment. *Beta*-lactamases are one of the most clinically and economically relevant ARGs, with *bla*_*TEM*_ among the earliest described beta-lactamase gene (Brusetti et al., 2008). *Beta*-lactamases by weight represents two thirds of total antibiotics administered to humans, therefore this has resulted in increased development and spread of bacterial resistance against these antibiotics (Anand et al., 2016). A study carried out by Zhang et al. (2019) on the dissemination of ARGs from manure treated soils to lettuce showed the ability of the plant tissues to take up ARGs, the rhizosphere of the lettuce harboring the most ARGs compared to the leaf and phyllosphere because of its direct contact with the soil, this presents a serious public health issue. With the increased consumption of raw and minimally processed foods this could result in transfer of ARGs to human commensal and pathogens.

## 5 Conclusion

Antibiotic resistance genes are not recognized as environmental contaminants in Botswana, wastewater effluent use remains unregulated which poses serious threat to public health. It is therefore imperative that research on antibiotic resistance and dissemination from wastewater treatment plants to agricultural environments is prioritized. This study shows that irrigation with wastewater effluent significantly changes the bacterial community profile in soil, potentially introduces ARGs into the soil and subsequently into fresh vegetable produce. This study supports other studies around the world that highlights the potential dissemination of ARGs from effluent to agricultural soils and vegetable crops. The government of Botswana has implemented an irrigation scheme that aims to use effluent for irrigation of vegetable crops to empower horticulture farmers and improve food security. However, without any antimicrobial resistance surveillance systems in place, the risk of potentially disseminating antibiotic resistance and pathogenic bacteria through the food chain remains. Considering the overall occurrence and diversity of antibiotic resistance determinants in agricultural settings and adding to the increase in consumption of raw vegetables, it is critical to put in place mitigation measures to reduce the risk of transmission of microbial infectious diseases. Hence this study has shed crucial findings on the impact of wastewater effluent irrigation in the spread of ARB and ARGs in agro-systems.

## Declarations

### Author contribution statement

Teddie O. Rahube: Conceived and designed the experiments; contributed reagents; materials; analysis tools; wrote the paper.

Onthatile Onalenna: Performed the experiments; analysed and interpreted the data; contributed reagents, materials, analysis tools; wrote the paper.

### Funding statement

This work was supported by Botswana International University of Science & Technology (Grant No: R0004).

### Data availability statement

Data associated with this study has been deposited at the NCBI SRA under the Accession number: PRJNA797192.

### Declaration of interests statement

The authors declare no conflict of interest.

### Additional information

No additional information is available for this paper.
